# Development and Validation of a Questionnaire to Assess Role Conflicts Among Interpreters Working With Refugee Clients: The Role Conflicts Questionnaire

**DOI:** 10.3389/ijph.2023.1605844

**Published:** 2023-09-11

**Authors:** Angelika Geiling, Laura Nohr, Caroline Meyer, Maria Böttche, Christine Knaevelsrud, Nadine Stammel

**Affiliations:** ^1^ Department of Education and Psychology, Division of Clinical Psychological Intervention, Freie Universität Berlin, Berlin, Germany; ^2^ Center ÜBERLEBEN, Berlin, Germany

**Keywords:** interpreters, role conflicts, expectations, emotional distress, refugee clients

## Abstract

**Objectives:** The aim of this study was to develop and validate a questionnaire to assess interpreters’ role conflicts and the challenging aspects within the triad of practitioner, interpreter and refugee client.

**Methods:** A questionnaire was developed based on previous literature. Its factor structure and construct validity were assessed in an online survey of 164 interpreters working with refugee clients. Psychological distress (BSI-18), work-related exhaustion (CBI), and secondary traumatic stress (ProQOL) were measured to test the questionnaire’s convergent validity.

**Results:** Exploratory structural equation modeling for categorical variables resulted in 23 items across four subscales. The scores of all subscales had good or excellent reliability (*ω* = 0.81 to *ω* = 0.93) and correlation analyses indicated convergent validity.

**Conclusion:** The final questionnaire (RoCo) showed four clearly interpretable subscales and may help to identify emotional distress due to role conflicts among interpreters. Future studies should validate the questionnaire in different samples.

## Introduction

In public service interpreting, interpreters usually work within a triad consisting of practitioner, interpreter, and client. They translate between two languages and mediate cultural codes [1], thus often embodying the role of a language and cultural mediator between practitioner and client. Generally, interpreters are required to follow principles such as confidentiality, impartiality, and accuracy [2, 3]. In many contexts, however, the role of the interpreter is not clearly defined, which leads to confusion regarding the roles of the triad members.

Working with refugee clients can be especially challenging because interpreters are confronted with clients’ stressful situations, such as insecure living situations and traumatization [4, 5]. In this regard, adherence to principles such as impartiality may conflict with clients’ expectations, for instance when clients ask interpreters for help [6].

So far, the distress experienced by interpreters due to their role within the triad has mainly been described in qualitative studies. Research has identified four overarching stressful role dynamics among interpreters working in triads: First, predominantly qualitative studies have frequently addressed emotional distress in the relationship with the client, especially regarding interpreters’ confrontation with traumatic content [6–9]. For instance, interpreters have reported feeling overwhelmed during appointments [8], difficulties in remaining emotionally detached [6], and difficulties in controlling their own emotional reactions [9]. Moreover, quantitative studies showed that interpreters can experience secondary traumatic distress (STS) [10, 11], which refers to the distress resulting from the work with traumatized clients [12]. Besides the emotional distress due to traumatic content, interpreters can be faced with the dilemma of feeling the need to comfort clients during sessions [6, 8] despite this not being part of their role.

Second, interpreters’ relationship with the practitioner can lead to further distress. For instance, research has shown that interpreters may perceive a lack of acknowledgement or respect from practitioners [8, 13, 14]. Indeed, several qualitative studies reported that interpreters felt that they were merely seen as a technical tool and thus felt devalued, for example, regarding their experience and knowledge [13, 15, 16].

Third, one of the most frequently discussed aspects of an interpreter’s work is the clarification of the interpreter’s role and the associated tasks. Previous research revealed that practitioners and clients have hugely varying expectations regarding the roles of interpreters, ranging from a perception of interpreters as cultural brokers to patient advocates, mediators, and basically invisible translators [17, 18]. Especially with regard to the client’s expectations, interpreters were found to be under increased pressure when they feel that clients have expectations beyond interpreting [4, 6, 14], such as translating documents and providing help regarding housing. Principles such as neutrality and impartiality, which are prerequisites for interpreting, were also sometimes experienced as contradicting cultural norms, for instance, when interpreters declined personal invitations from clients [15]. As coping strategies, interpreters generally mentioned setting clear boundaries and trying to accept the limitations of their role [9].

Finally, in the process of interpreting between practitioner and client, the relationship with the client can shift from the practitioner to the interpreter, in terms of more eye contact or body language towards the interpreter [19], such as at the beginning of the triadic relationship [15] or in crisis situations as the interpreter is more easily accessible for the client [19]. In general, interpreters reported that it is challenging to fulfill the expectations of both sides [6, 20] and pointed out their sensitive position between client and practitioner [6, 13].

Previous qualitative studies have indicated a relationship between role dynamics and psychological distress, especially work-related distress, among interpreters [8, 14, 15]. However, the potential influence of role dynamics has not yet been systematically assessed, and the association between role conflicts and mental health has consequently not been adequately examined. To date, there is no questionnaire that quantifies role conflicts among interpreters. The first aim of this research was therefore to develop and validate a questionnaire that measures role conflicts and challenging aspects of interpreters’ relationships within the triad of interpreter, practitioner, and client. The second aim was to investigate its convergent construct validity by exploring possible relationships with psychological and work-related distress.

## Methods

### Sample and Sampling

A Germany-wide anonymous online survey was conducted using the online survey platform Unipark EFS Survey [21]. The sample was recruited *via* opportunity and snowball sampling at psychosocial and public institutions working with interpreters for refugees. Inclusion criteria for participation were 1) age ≥ 18 years and 2) being paid for interpreting spoken languages for refugee clients. Interpreters could participate between April 2019 and July 2019. Participants were informed that the aim of the survey was to investigate the mental health of interpreters and possible helpful and difficult aspects of their work. All participants provided written informed consent prior to participation and were informed that they could withdraw from the survey at any time. The study was approved by the Ethics Committee of the Freie Universität Berlin. Overall, 291 participants gave their consent to participate, of whom *N* = 164 participants were included in the analysis. Further details regarding the recruitment process are provided elsewhere [22].

### Development of the Role Conflicts Questionnaire

The development of the Role Conflicts Questionnaire - German Version (RoCo) began with a non-systematic search of the literature on interpreters’ roles and perceived difficulties of interpreting in different work settings. Based on previous findings regarding interpreters’ roles and their role conflicts in working environments, a working definition of role conflicts was developed, comprising four areas: 1) emotional reactions due to the client’s stories, 2) difficulties in the relationship with practitioners, 3) lack of clarification of the interpreter’s role, and 4) difficult dynamics within the triad. The first author generated 32 items in the form of statements regarding the understanding of one’s role and personally perceived difficulties. Subsequently, the content and wording of the items were discussed and revised with an experienced researcher and clinical psychologist for refugee care. The 32 items were then grouped into four subscales based on the working definition. Next, the items were randomized and eight experienced refugee care professionals from a specialized center for the treatment of war and torture victims (*n* = 2 staff contact persons for interpreters, *n* = 2 interpreters, *n* = 1 psychotherapist, *n* = 3 researchers) were asked to designate the allocation of each item to one of the four predefined subscales. The results of the ratings and item allocation were discussed by NS and AG and the number of items was reduced by five items based on the raters’ comments and inconclusive assignment to the subscales. The survey was conducted using the final questionnaire with 27 items. Items are rated on a 7-point Likert-type scale [1–7] from 1 = “not true at all” through 4 = “partially true” to 7 = “completely true.” Depending on each respondent’s main work setting, the word “practitioner” was replaced accordingly (psychotherapy: psychotherapist, counselling: counsellor; authorities: authority employee; medical settings: doctor; others: practitioner).

### Survey

First, we gathered sociodemographic characteristics (e.g., gender, age, flight experiences). Interpreters had to indicate one of five main work settings: 1) psychotherapy, 2) psychosocial counselling (i.e., drug counselling, family counselling), 3) medical setting (i.e., hospital or doctor’s office), 4) authorities (i.e., German Federal Office for Migration and Refugees), court, police, social services, job center, or 5) other setting. Subsequently, we asked about details of participants’ work as interpreters in their main work setting. To assess the convergent construct validity of the newly developed RoCo, three additional questionnaires were applied:

Psychological distress was measured using the Brief Symptom Inventory 18 (BSI-18; [23, 24]), which assesses symptoms of depression, anxiety, and somatization with six items each. Items are rated on a 5-point Likert-type scale ranging from 0 = “not at all” to 4 = “extremely.” In the current analysis, we calculated the General Severity Index (GSI) as a global indicator of psychological distress, adding all 18 items together to form a sum score (0–72). Higher scores indicate higher distress. The internal consistency in the current sample was McDonald’s *⍵* = 0.92, Cronbach’s *α* = 0.91.

Work-related exhaustion was assessed using one of three subscales of the Copenhagen Burnout Questionnaire (CBI; [25]), referred to in the present study as CBI-work-related. The subscale comprises seven items assessing the extent to which respondents associate their work with feelings of exhaustion. The items are rated on a 5-point Likert-type scale from 1 = “to a very low degree” or “never” to 5 = “to a very high degree” or “always.” The Likert-type scale was converted (1 = 0; 2 = 25; 3 = 50; 4 = 75; 5 = 100) and the mean score was calculated as the total score. Scores could range from 0 to 100 with higher scores indicating higher levels of work-related exhaustion. In the current sample, internal consistency was *⍵* = 0.89, *α* = 0.86.

Lastly, secondary traumatic stress experienced by the participants was assessed using the respective subscale of the Professional Quality of Life Scale (ProQOL; [26]). The ProQOL-STS consists of ten items that are rated on a 5-point Likert-type scale ranging from 1 = “never” to 5 = “very often.” To improve the fit of the items to the work context of professional interpreters, the word “help” was replaced with “interpret for” (as proposed by the manual). Scores could range from 10 to 50 with higher scores indicating higher levels of STS. For validity testing, the sum score of the raw scores was calculated. The internal consistency in the present sample was *⍵* = 0.84, *α* = 0.81 [24].

With the exception of the BSI-18, all questionnaires were not originally published in German. Therefore, we applied the back-translation procedure to ensure the quality of the German translations [25]. The CBI and ProQOL have not been validated in German language and context. To establish a minimum of reliability, we applied exploratory factor analyses (EFA) with satisfactory findings. Detailed information on the results can be found in the Supplementary Material SE.

### Missing Data and Multiple Imputation

The final data set of the RoCo (*N* = 164) revealed a number of missing values on the item level, ranging from 0.6% (items 2, 4, 10, 19, 21, and 25) to 3% (item 14). We analyzed missing data of the RoCo, and missing values were replaced using multiple imputation. First, we tested the underlying missing data mechanism. Little’s MCAR (Missing Completely at Random) test was not significant, *Χ* ^*2*^ = 531.77, *df* = 560, *p* = 0.79, indicating that data were missing completely at random [27]. Further, influx and outflux of each item were checked to assess their usefulness in the context of the imputation model [27]. Both of these indicators are summaries of missing data patterns and showed favorable scores for multiple imputation [27]. In line with recent recommendations [28–30], we applied multiple imputation at the item level using predictive mean matching (PMM). PMM was conducted using the mice package in R [31] to generate 50 imputed data sets. The quality of imputations was examined *post hoc* using density plots [32]. Overall, the imputation algorithms were stable. Finally, for each analysis, the individually estimated parameters were pooled into a single set of results using Rubin’s rules [33].

### Statistical Analyses

Descriptive analyses were conducted for all variables of interest. As a first step of psychometric validation, exploratory structural equation modeling (ESEM) was applied to identify a reasonable factor structure of the RoCo. Statistical assumptions for factor analyses were examined [34]. Due to the non-normal distribution of the data, a weighted least squares mean and variance adjusted (WLSMV) estimator was used for categorical data [35, 36]. Oblique rotation was applied in all ESEMs since the latent factors of interpreters’ role conflicts were expected to be intercorrelated [35]. To identify an adequate number of factors, the Kaiser-Guttmann criterion (Eigenvalues > 1), parallel analysis, the goodness-of-fit indices, and the interpretability of the factor solution were considered. In the next step, items were selected and eliminated successively. Single items were evaluated based on factor loadings and cross-loadings [35], with items with a loading lower than 0.3 being excluded. After selecting a model, the reliability and validity of the identified factors were evaluated by calculating internal consistency for each factor, reporting McDonald’s omega as a model-based estimate of reliability [37]. Furthermore, the following fit indices and cut-off scores were considered to explore the model fit of each model [38]: >0.95 for the comparative fit index (CFI) and the Tucker-Lewis index (TLI), <.06 for the root mean square error of approximation (RMSEA), and <0.08 for the standardized root mean square residual (SRMR). Further, results of χ^2^ tests were considered even though they are based on specific distributional assumptions and sensitive to rejecting the null hypothesis [35]. Additionally, we correlated the identified subscales with gender and work experience, and tested convergent validity using bivariate correlations with psychological distress, work-related exhaustion, and STS. Therefore, a mean score for each subscale of the RoCo was calculated. Statistical analyses were conducted using the software R 4.2.1 [39] with the software packages mice [31] and miceadds [40] as well as the software program Mplus 8.1 [41].

## Results

### Sample Description

The final sample consisted of *N* = 164 interpreters (*n* = 115 females, 70.1%). Levels of psychological distress were significantly higher than in a German representative sample [42]. Furthermore, lower scores of work-related exhaustion were reported in the present sample compared to similar professions [43] and similar high scores of STS were reported as in a previous interpreter sample [44]. See Table 1 for detailed characteristics of the sample.

**TABLE 1:** Sample characteristics (Germany, April 2019 until July 2019).

	*n*	**%**	Mean	SD	Range
Sociodemographic Variables					
Gender: Female	115	70.1	—	—	—
Age, in years	—	—	38.84	12.35	18–71
Years of education	—	—	16.80	3.42	6–26
Ever fled or displaced, yes	45	27.4	—	—	
Degree in interpreting (university or college)	24	14.6	—	—	
Work experience in years^a^	—	—	5.17	5.97	0–30
Employment situation^a^					
Freelancer	108	65.9	—	—	—
Employed	37	22.6	—	—	—
Both	19	11.6	—	—	—
Main work setting					
Psychotherapy	38	23.2	—	—	—
Authorities	59	36.0	—	—	—
Medical	22	13.4	—	—	—
Counselling	39	23.8	—	—	—
Other Setting	6	3.7	—	—	—
Psychological and work-related distress					
Psychological distress (BSI-18 GSI)	—	—	9.02	9.07	0–43
Secondary traumatic stress (ProQOL-STS)	—	—	18.90	5.78	10–37
Work-related exhaustion (CBI - work-related)	—	—	25.78	18.31	0–93

Not*e*. BSI-18 GSI: Brief Symptom Inventory-18 General Severity Index; CBI, work-related: Copenhagen Burnout Questionnaire, subscale: work-related; ProQOL-STS: professional quality of life, subscale Secondary traumatic stress (raw scores).

^a^ In main work setting.

### Exploratory Factor Analysis

The Kaiser-Meyer-Olkin measure of sampling accuracy indicated an adequate sample size for factor analysis (KMO = 0.81) and Bartlett’s test of sphericity was significant [*χ* ^2^ (351) = 2168.669; *p* < 0.001]. The ESEM including all 27 items revealed four factors with eigenvalues >1, thus favoring a four-factor solution based on the Kaiser-Guttmann criterion. The scree plot suggested either a two- or a four-factor solution while the parallel test favored a four-factor solution. Considering all applied criteria, a four-factor solution was selected. To improve interpretability, items were removed in a stepwise procedure following the rules outlined by Rosellini and Brown [35]. First, ESEM was conducted with the full questionnaire (Model 1). In this analysis, item 6 showed low factor loadings in general and similarly high loadings on both F1 and F4 (0.36 and 0.32, respectively) and was therefore removed. ESEM was conducted again with 26 items (Model 2). In this analysis, item 16 showed factor loadings ≤0.3 for all four factors and was also excluded. Next, item 24 was deleted due to almost similarly high loadings on F3 (0.56) and F4 (−0.51) (model 4). Lastly, item 23 showed similarly high loadings on F2 (−0.38) and F3 (0.47) and was therefore deleted. In the resulting model 5, a simple structure was reached, such that all items had high loadings on one factor and substantially lower loadings on the other factors; we therefore stopped item evaluation and reanalysis at this point. Goodness-of-fit indices for all five models are shown in Table 2. All the indices were close to or above the suggested cut-offs for good model fit.

**TABLE 2:** Goodness-of-fit indices for Model 1 to Model 5 (Germany, April 2019 until July 2019).

	WLSMV- *χ* ^*2*^	*df*	CFI	TLI	RMSEA	SRMR
Model 1: original (27 items)	525.097	249	0.956	0.938	0.082	0.048
Model 2: 26 items	494.936	227	0.957	0.939	0.085	0.046
Model 3: 25 items	483.291	206	0.956	0.935	0.091	0.045
Model 4: 24 items	353.060	186	0.973	0.959	0.074	0.037
**Model 5: 23 items**	**313.116**	**167**	**0.976**	**0.964**	**0.073**	**0.035**

Note. *N* = 164. WLSMV: weighted least squares with mean and variance adjustment; CFI, comparative fit index; TLI, Tucker Lewis index; RMSEA, root mean square error of approximation; SRMR, standardized root mean square residual. Model 5 (in bold) was chosen as the final model. Model 1 (27 items); Model 2 (26 items, item 6 removed), Model 3 (25 items, items 6 and 16 removed), Model 4 (24 items, items 6, 16, and 23 removed) and Model 5 (23 items, items 6, 16, 22 and 23 removed).

Model 5 was chosen as the final model. Its four-factor structure corresponds to the four overarching themes introduced in the working definition. Factor loadings of the items are shown in Table 3. The first factor reflects emotional and cognitive difficulties in setting boundaries in the relationship between interpreter and client (e.g., item 5, “It is difficult for me to distance myself mentally from the clients after the appointments”) and was named “Lack of emotional boundaries between interpreter and client.” Higher scores on this subscale reflect a higher lack of emotional boundaries between interpreter and client. The second factor, “Devaluation by practitioners,” reflects problems that primarily emerge in the relationship between interpreter and practitioner (e.g., item 10, “I do not feel appreciated by practitioners”). Regarding this subscale, higher scores reflect a higher perceived devaluation by practitioners. The third factor, “Perceived formal framework of the interpreter’s role,” reflects the perception of the formal framework of the interpreter’s role (e.g., item 15 “My role as an interpreter is clearly defined by practitioners”). The higher the score on this subscale, the clearer the formal framework of the interpreter’s role is perceived by the respondent. The fourth factor, “Emotional distress due to the role within the triad,” reflects dynamic problems that emerge from the triadic relationship between interpreter, practitioner, and client (e.g., item 20, “I am worried that I disrupt the relationship between clients and practitioners”). Higher scores on this subscale reflect higher distress due to the interpreter’s role within the triad. The scores of all factors in the present sample showed good to excellent reliability (*ω* = 0.81–*ω* = 0.93). Inter-factor correlations are shown in Table 4 and indicate that factors are not independent from each other.

**TABLE 3:** Exploratory factor analysis. Factor loadings of Model 5 (Germany, April 2019 until July 2019).

	Item description	Mean	SD	Factor loadings			
				F1	F2	F3	F4
Factor 1: Lack of emotional boundaries between interpreter and client							
Item 1	I feel the need to calm clients down during the appointment.	3.92	1.79	**0.39**	0.24	0.22	−0.00
Item 2	I have to cry during appointments with clients.	1.83	1.27	**0.63**	0.15	0.04	−0.02
Item 3	I have to think about the clients for a long time after the appointments.	3.24	1.69	**0.85**	0.08	0.04	−0.06
Item 4	I feel emotionally distressed after the appointments.	3.01	1.57	**0.83**	0.12	0.08	−0.04
Item 5	It is difficult for me to distance myself mentally from the clients after the appointments.	2.57	1.56	**0.98**	0.00	−0.07	−0.09
Item 6	It is difficult for me to set emotional boundaries between myself and the clients during the appointment.	2.46	1.55	**0.82**	−0.17	−0.04	0.22
Item 7	It is difficult for me to set emotional boundaries between myself and the clients after the appointment.	2.23	1.40	**0.85**	−0.07	−0.01	0.14
Factor 2: Devaluation by practitioners							
Item 8	I am not treated as an equal communication partner by practitioners.	2.19	1.68	0.02	**0.67**	0.06	0.10
Item 9	I have the impression that practitioners evaluate my work unfairly.	1.69	1.32	−0.03	**0.92**	0.01	0.00
Item 10	I do not feel appreciated by practitioners.	1.71	1.42	−0.05	**0.95**	0.03	−0.00
Item 11	I have the impression that practitioners speak to me in a derogatory tone.	1.49	1.04	0.03	**0.84**	−0.11	0.11
Item 12	I have the impression that practitioners only see me as a technical tool.	2.01	1.56	0.08	**0.83**	0.02	−0.03
Item 13	I have the impression that practitioners attribute misunderstandings to poor interpreting.	1.91	1.48	0.11	**0.68**	−0.22	0.04
Factor 3: Perceived formal framework of the interpreter’s role							
Item 14	My neutral role as an interpreter is compatible with the client’s cultural values.	4.01	2.13	0.08	−0.11	**0.55**	−0.06
Item 15	My role as an interpreter is clearly defined by practitioners.	4.90	1.84	−0.02	−0.00	**0.86**	0.06
Item 16	My job as an interpreter is clearly defined in advance by practitioners.	4.93	1.72	0.01	−0.05	**0.85**	0.09
Item 17	I know what clients expect from me as an interpreter.	5.10	1.67	−0.10	0.19	**0.52**	−0.26
Item 18	The rules regarding my job as an interpreter were conveyed to me by practitioners.	4.51	2.07	0.12	−0.09	**0.50**	−0.13
Item 19	Clients understand my neutral role.	4.15	1.71	−0.08	−0.13	**0.41**	−0.22
Factor 4: Emotional distress due to the role within the triad							
Item 20	I am worried that I disrupt the relationship between clients and practitioners.	2.05	1.35	−0.01	0.01	−0.01	**0.96**
Item 21	I am worried that I am an obstacle to the relationship between clients and practitioners.	2.03	1.45	0.02	0.03	0.04	**0.89**
Item 22	I am worried that clients have a closer relationship with me than with practitioners.	2.85	1.80	0.10	0.28	.04	**0.54**
Item 23	I feel distressed when clients and practitioners have conflicting needs during the appointments.	3.04	1.84	0.28	0.18	0.03	**0.49**
		***ω***		**0.92**	**0.93**	**0.81**	**0.90**
		***R*** ^***2***^		**0.67**	**0.73**	**0.47**	**0.70**

Note. *N* = 164. Factors were extracted using a WLSMV estimator with oblique rotation; factor loadings ≥ 0.30 are printed in bold. *ω* = McDonald’s Omega; R^2^ = coefficient of determination. Deleted items: former item 6: I am worried that I may encounter clients outside the appointment (Factor 1: Lack of emotional boundaries between interpreter and client); former item 16: As an interpreter, I have limited scope to act (Factor 3: Perceived formal framework of the interpreter’s role); former item 20: Practitioners understand my neutral role (Factor 3: Perceived formal framework of the interpreter’s role); former item 21: Clients understand that I have to keep a professional distance from them (Factor 3: Perceived formal framework of the interpreter’s role).

**TABLE 4:** Inter-factor correlations in Model 5 (23 items) (Germany, April 2019 until July 2019).

	Mean	SD	F1	F2	F3
F1	2.73	1.21	—		
F2	1.81	1.11	0.59	—	
F3	4.66	1.16	−0.31	−0.44	—
F4	2.43	1.31	0.16	0.25	−0.12

Note. F1: Lack of emotional boundaries between interpreter and client; F2: Devaluation by practitioners; F3: Perceived formal framework of the interpreter’s role; F4: Emotional distress due to the role within the triad.

### Assessment of Convergent Validity

To assess convergent validity, the scores of the four subscales were correlated with psychological distress in general (BSI-GSI), in the workplace (CBI, subscale work-related exhaustion), and STS (ProQOL). The three factors that measure distress due to role conflicts in the interpreting setting (F1, F2, and F4) showed significant positive correlations with measures of mental distress in general (*r* = 0.27 – *r* = 0.50) and in the workplace (*r* = 0.32 – *r* = 0.50), and with STS (*r* = 0.34 – *r* = 0.44). In contrast, as expected, the factor measuring the perceived formal framework of the interpreter’s role (F3) showed significant negative correlations with measures of mental distress in general and in the workplace, and with STS (*r* = −0.11 – *r* = −0.21). See Table 5 for details.

**TABLE 5:** Correlations between factors and external variables for Model 5 (23 items) (Germany, April 2019 until July 2019).

	F1	F2	F3	F4
Gender: male	−0.12***	0.02	0.03*	−0.01
Work experience in years^a^	−0.26***	−0.02***	0.10***	−0.17***
Psychological distress (BSI-18 GSI)	0.46***	0.27***	−0.19***	0.50***
Secondary traumatic stress (ProQOL-STS)	0.44***	0.34***	−0.21***	0.46***
Work-related exhaustion (CBI—work-related)	0.50***	0.32***	−0.11***	0.44***

Note. ***p < 0.001; *p < 0.05.

^a^ In main work setting; BSI-18 GSI: Brief Symptom Inventory-18 General Severity Index; CBI, work-related: Copenhagen Burnout Questionnaire, subscale: work-related; ProQOL-STS, professional quality of life, subscale Secondary traumatic stress (raw scores). F1: Lack of emotional boundaries between interpreter and client; F2: Devaluation by practitioners; F3: Perceived formal framework of the interpreter’s role; F4: Emotional distress due to the role within the triad.

## Discussion

The aim of the present study was to develop and evaluate a questionnaire assessing role conflicts and challenging aspects of interpreting for refugee clients. For this purpose, a newly developed questionnaire was psychometrically tested, and the convergent validity of the subscales of the questionnaire was assessed. The results of the ESEM revealed a questionnaire with four subscales assessing various challenging role conflicts of interpreters within the triad consisting of practitioner, client, and interpreter. The scores of the final RoCo questionnaire showed excellent psychometric properties and a preliminary assessment of convergent validity revealed promising findings.

### Evaluation of the Questionnaire

The questionnaire was developed based on qualitative reports of interpreters working with refugee and migrated clients and a preliminary working definition of the role of interpreters. After deleting four items due to low factor loadings and cross-loadings in the ESEM, 23 items across four subscales resulted: 1) lack of emotional boundaries between interpreter and client, 2) devaluation by practitioners, 3) perceived formal framework of the interpreter’s role, and 4) emotional distress due to the role within the triad. The first subscale includes items on rumination and distress due to hearing and interpreting the client’s stories, which is reflected in interpreters’ reported difficulties in handling their own emotions during or after appointments [7, 9]. The second subscale focuses on feeling devalued in the relationship with practitioners, for example, due a lack of appreciation of the interpreter’s work [8, 15]. Furthermore, this subscale includes the increasingly criticized perception of interpreters as a black box or technical tool [45] as a possible cause of distress. The third subscale comprises the interpreters’ perception regarding the clarification of their role. As such, it reflects the lack of formal standards for the role of interpreters, which can range from mere translation to cultural mediation [17, 18]. The fourth subscale includes the dynamics between all three parties within the triad and addresses, for example, interpreters’ concerns about being an obstacle to the relationship between practitioner and client. This subscale therefore corresponds well to the experiences reported in the literature, such as interpreters’ feelings of distress when they notice difficult situations in the relationship between client and practitioner [14, 15]. However, the fourth factor contains only four items and might benefit from revision, for example, by adding items pertaining to further difficult dynamics within the triadic relationship. In consequence, the four areas described in the working definition and in qualitative studies are in line with the results of the ESEM. Overall, the analysis revealed factors that can be clearly distinguished from each other and show high factor loadings of the items on the respective factor.

The convergent construct validation was exploratory using questionnaires related to psychological distress and work-related constructs (work-related exhaustion and STS). As expected, the established measures of psychological and work-related distress showed positive correlations with the first (lack of emotional boundaries between interpreter and client), second (devaluation by practitioners) and fourth subscale (emotional distress due to the role within the triad) of the RoCo and negative correlations with the third subscale (perceived formal framework of the interpreter’s role). The RoCo therefore captures an independent construct with facets of psychological and work-related distress.

Furthermore, it should be noted that the RoCo was evaluated in the context of working with refugee clients. The first subscale in particular might be especially relevant for working with traumatized (refugee) clients, as it focuses on the emotional distress due to the client’s stories. However, the questionnaire may also be applied more generally to interpreters working with culturally and linguistically diverse clients, as studies have highlighted a lack of role clarification, for instance, for interpreting contexts beyond that of refugee clients [17, 18]. Therefore, we suggest applying the questionnaire in various settings and with all types of clients to gain a better understanding of interpreters’ challenging work and role conflicts within the triad.

### Strengths and Limitations

The RoCo is the first questionnaire to systematically assess role conflicts for interpreters in the triad between interpreter, practitioner, and client.

Several limitations must be considered. In the present study, a convenience sample of interpreters was recruited at various locations, and the sample is therefore not representative for interpreters in general. Nevertheless, a reasonably high sample size was achieved for the online survey, and we applied the questionnaire in various work settings. Due to the small sample size per work setting, it was not possible to analyze the questionnaire separately for every work setting. Consequently, the sample of interpreters may have been heterogeneous in terms of the clarification of their role. The questionnaire is based on self-reporting only. In future studies observer ratings may help to contribute to a better comprehension of role conflicts. Moreover, we did not account for response bias or socially desirable responding. However, the online survey was completely anonymous which should have reduced the possibility of socially desirable responding. The questionnaires were not counterbalanced across participants. Additionally, we only explored convergent validity, further investigation of discriminant constructs needs to be addressed in the future. Overall, this is the first sample in which the RoCo is assessed. Therefore, future studies should further evaluate the factor structure using confirmatory factor analysis and examine the reliability and validity in an independent sample.

### Conclusion and Implications

This study took a first step to investigate and quantify role conflicts among interpreters. The first evaluation showed clearly interpretable subscales with high internal consistencies. Based on the present findings and the research to date, the questionnaire needs to be applied, further improved, and validated in more settings and languages. Further research on the relationships between socio-demographic, work-related variables, clinical disorders, and the subscales of the RoCo may contribute to a better understanding of role conflicts among interpreters and, furthermore, provide indications for future research on coping with role conflicts. Additionally, discriminant construct validation was not addressed in the current study and requires further investigation. By applying the questionnaire, we aim to facilitate a process of open communication between interpreters and practitioners regarding difficult situations and develop a mutual understanding among the three parties. Importantly, the questionnaire may point to relevant topics in terms of employer support for interpreters. Ideally, therefore, the RoCo will enhance and contribute to a functional work environment for interpreters in refugee care.
