# Supplementary material for: Development and Validation of a Questionnaire to Assess Role Conflicts Among Interpreters Working With Refugee Clients: The Role Conflicts Questionnaire
Source: Int J Public Health. 2023 Sep 11;68:1605844. doi: 10.3389/ijph.2023.1605844 (PMC10518398; doi:10.3389/ijph.2023.1605844)
Supplement: Supplementary file 1 [file DataSheet1.docx]

**Supplement**

- A: Original questionnaire (German version)
- B: Factor loadings for version with all 27 items
- C: Inter-item correlations
- D: Final version (German)
- E: EFA of CBI-work-related and ProQOL-STS

**Supplement A**

German Role Conflict questionnaire (original version)

| Item 1 | Ich habe das Bedürfnis, Klient*innen während des Termins zu beruhigen. |
| --- | --- |
| Item 2 | Ich muss während der Termine mit Klient*innen weinen. |
| Item 3 | Ich muss nach den Terminen lange an die Klient*innen denken. |
| Item 4 | Ich fühle mich nach den Terminen emotional belastet. |
| Item 5 | Mir fällt es schwer, nach den Terminen von den Klient*innen gedanklich Abstand zu nehmen. |
| Item 6 | Ich mache mir Sorgen, dass ich Klient*innen außerhalb des Termins treffen könnte. |
| Item 7 | Es fällt mir schwer, mich während des Termins emotional von Klient*innen abzugrenzen. |
| Item 8 | Es fällt mir schwer, mich nach dem Termin emotional von Klient*innen abzugrenzen. |
| Item 9 | Ich werde nicht als gleichwertige*r Kommunikationspartner*in von Auftraggebenden behandelt. |
| Item 10 | Ich habe den Eindruck, dass Auftraggebende meine Arbeit unfair bewerten. |
| Item 11 | Ich fühle mich von Auftraggebenden nicht wertgeschätzt. |
| Item 12 | Ich habe den Eindruck, Auftraggebende sprechen in einem herabwürdigenden Ton mit mir. |
| Item 13 | Ich habe den Eindruck, Auftraggebende sehen mich nur als technisches Werkzeug. |
| Item 14 | Ich habe den Eindruck, Auftraggebende führen Missverständnisse auf schlechtes Dolmetschen zurück. |
| Item 15 | Meine neutrale Rolle als Dolmetschende*r ist mit den kulturellen Werten der Klient*innen vereinbar. |
| Item 16 | Ich habe begrenzte Handlungsmöglichkeiten als Dolmetschende*r. |
| Item 17 | Meine Rolle als Dolmetschende*r ist klar durch Auftraggebende definiert. |
| Item 18 | Meine Aufgaben als Dolmetschende*r sind klar von Auftraggebenden vorgegeben. |
| Item 19 | Ich weiß, was Klient*innen von mir als Dolmetschende*r erwarten. |
| Item 20 | Die Regeln zu meiner Dolmetschendentätigkeit sind mir von Auftraggebenden vermittelt worden. |
| Item 21 | Klient*innen verstehen meine neutrale Rolle. |
| Item 22 | Auftraggebende verstehen meine neutrale Rolle. |
| Item 23 | Klient*innen verstehen, dass ich eine professionelle Distanz zu ihnen einhalten muss. |
| Item 24 | Ich habe Sorge, die Beziehung zwischen Klient*innen und Auftraggebenden zu stören. |
| Item 25 | Ich habe Sorge, ein Hindernis für die Beziehung zwischen Klient*innen und Auftraggebenden zu sein. |
| Item 26 | Ich habe Sorge, dass Klient*innen eine engere Beziehung zu mir haben als zu Auftraggebenden. |
| Item 27 | Ich bin belastet, wenn es widersprüchliche Bedürfnisse von Klient*innen und Auftraggebenden während der Termine gibt. |

**Supplement B**

Exploratory factor analysis for the original version of the questionnaire (27 items)

|  | Item description | Mean | SD | | Factor loadings | | | |
| --- | --- | --- | --- | --- | --- | --- | --- | --- |
|  |  |  |  | F1 | | F2 | F3 | F4 |
| **Factor 1: Lack of emotional boundaries between interpreter and client** | | | | | | | | |
| Item 1 | I feel the need to calm clients down during the appointment. | 3.92 | 1.79 | **.37** | | .24 | .20 | .03 |
| Item 2 | I have to cry during appointments with clients. | 1.83 | 1.27 | **.64** | | .14 | .05 | -.04 |
| Item 3 | I have to think about the clients for a long time after the appointments. | 3.24 | 1.69 | **.83** | | .07 | -.01 | -.02 |
| Item 4 | I feel emotionally distressed after the appointments. | 3.01 | 1.57 | **.82** | | .13 | .07 | -.03 |
| Item 5 | It is difficult for me to distance myself mentally from the clients after the appointments. | 2.57 | 1.56 | **.97** | | .00 | -.08 | -.09 |
| Item 6 | I am worried that I may encounter clients outside the appointment. | 2.15 | 1.64 | **.36** | | .12 | .10 | **.31** |
| Item 7 | It is difficult for me to set emotional boundaries between myself and the clients during the appointment. | 2.46 | 1.55 | **.84** | | -.14 | -.02 | .19 |
| Item 8 | It is difficult for me to set emotional boundaries between myself and the clients after the appointment. | 2.23 | 1.40 | **.86** | | -.04 | -.01 | .13 |
| **Factor 2: Devaluation by practitioners** | | | | | | | | |
| Item 9 | I am not treated as an equal communication partner by practitioners. | 2.19 | 1.68 | .02 | | **.68** | .07 | .10 |
| Item 10 | I have the impression that practitioners evaluate my work unfairly. | 1.69 | 1.32 | -.04 | | **.92** | .01 | .00 |
| Item 11 | I do not feel appreciated by practitioners. | 1.71 | 1.42 | -.05 | | **.96** | .05 | -.02 |
| Item 12 | I have the impression that practitioners speak to me in a derogatory tone. | 1.49 | 1.04 | .06 | | **.85** | -.09 | .06 |
| Item 13 | I have the impression that practitioners only see me as a technical tool. | 2.01 | 1.56 | .06 | | **.84** | .03 | -.04 |
| Item 14 | I have the impression that practitioners attribute misunderstandings to poor interpreting. | 1.91 | 1.48 | .13 | | **.69** | -.19 | .00 |
| **Factor 3: Perceived formal framework of the interpreter’s role** | | | | | | | | |
| Item 15 | My neutral role as an interpreter is compatible with the client’s cultural values. | 4.01 | 2.13 | .12 | | -.05 | **.60** | -.12 |
| Item 16 | As an interpreter, I have limited scope to act. | 4.24 | 2.02 | .16 | | .21 | **.30** | -.10 |
| Item 17 | My role as an interpreter is clearly defined by practitioners. | 4.9 | 1.84 | -.09 | | .03 | **.83** | .17 |
| Item 18 | My job as an interpreter is clearly defined in advance by practitioners. | 4.93 | 1.72 | -.09 | | -.04 | **.82** | .26 |
| Item 19 | I know what clients expect from me as an interpreter. | 5.1 | 1.67 | -.15 | | .21 | **.49** | -.22 |
| Item 20 | The rules regarding my job as an interpreter were conveyed to me by practitioners. | 4.51 | 2.07 | .09 | | -.11 | **.49** | -.05 |
| Item 21 | Clients understand my neutral role. | 4.15 | 1.71 | .05 | | -.08 | **.60** | **-.46** |
| Item 22 | Practitioners understand my neutral role. | 5.46 | 1.58 | .06 | | **-.37** | **.46** | -.12 |
| Item 23 | Clients understand that I have to keep a professional distance from them. | 4.15 | 1.75 | .10 | | -.12 | **.56** | **-.50** |
| **Factor 4: Emotional distress due to the role within the triad** | | | | | | | | |
| Item 24 | I am worried that I disrupt the relationship between clients and practitioners. | 2.05 | 1.35 | .10 | | .03 | .05 | **.89** |
| Item 25 | I am worried that I am an obstacle to the relationship between clients and practitioners. | 2.03 | 1.45 | .11 | | .03 | .09 | **.86** |
| Item 26 | I am worried that clients have a closer relationship with me than with practitioners. | 2.85 | 1.8 | .17 | | **.30** | .07 | **.50** |
| Item 27 | I feel distressed when clients and practitioners have conflicting needs during the appointments. | 3.04 | 1.84 | **.32** | | .20 | .02 | **.46** |
|  |  |  |  |  | |  |  |  |

*Note. N* = 164. Factors were extracted using a WLSMV estimator with oblique rotation; factor loadings ≥ .30 are printed in bold.

**Supplement C**

*Bivariate correlations between all items of the final version of the RoCo*

| **Item No.** | **1** | **2** | **3** | **4** | **5** | **6** | **7** | **8** | **9** | **10** | **11** | **12** | **13** | **14** | **15** | **16** | **17** | **18** | **19** | **20** | **21** | **22** |
| --- | --- | --- | --- | --- | --- | --- | --- | --- | --- | --- | --- | --- | --- | --- | --- | --- | --- | --- | --- | --- | --- | --- |
| *Factor 1* |  |  |  |  |  |  |  |  |  |  |  |  |  |  |  |  |  |  |  |  |  |  |
| Item 1 | - |  |  |  |  |  |  |  |  |  |  |  |  |  |  |  |  |  |  |  |  |  |
| Item 2 | .47 | - |  |  |  |  |  |  |  |  |  |  |  |  |  |  |  |  |  |  |  |  |
| Item 3 | .49 | .55 | - |  |  |  |  |  |  |  |  |  |  |  |  |  |  |  |  |  |  |  |
| Item 4 | .40 | .61 | .73 | - |  |  |  |  |  |  |  |  |  |  |  |  |  |  |  |  |  |  |
| Item 5 | .38 | .66 | .83 | .81 | - |  |  |  |  |  |  |  |  |  |  |  |  |  |  |  |  |  |
| Item 6 | .38 | .56 | .64 | .67 | .76 | - |  |  |  |  |  |  |  |  |  |  |  |  |  |  |  |  |
| Item 7 | .30 | .55 | .71 | .74 | .80 | .85 | - |  |  |  |  |  |  |  |  |  |  |  |  |  |  |  |
| *Factor 2* |  |  |  |  |  |  |  |  |  |  |  |  |  |  |  |  |  |  |  |  |  |  |
| Item 8 | .26 | .29 | .29 | .30 | .25 | .25 | .29 | - |  |  |  |  |  |  |  |  |  |  |  |  |  |  |
| Item 9 | .35 | .26 | .23 | .38 | .22 | .17 | .30 | .69 | - |  |  |  |  |  |  |  |  |  |  |  |  |  |
| Item 10 | .33 | .31 | .26 | .33 | .22 | .14 | .28 | .64 | .86 | - |  |  |  |  |  |  |  |  |  |  |  |  |
| Item 11 | .29 | .39 | .31 | .39 | .35 | .25 | .30 | .64 | .80 | .84 | - |  |  |  |  |  |  |  |  |  |  |  |
| Item 12 | .27 | .40 | .34 | .34 | .30 | .26 | .32 | .58 | .77 | .77 | .78 | - |  |  |  |  |  |  |  |  |  |  |
| Item 13 | .27 | .28 | .36 | .33 | .34 | .25 | .32 | .50 | .65 | .68 | .76 | .67 | - |  |  |  |  |  |  |  |  |  |
| *Factor 3* |  |  |  |  |  |  |  |  |  |  |  |  |  |  |  |  |  |  |  |  |  |  |
| Item 14 | .13 | .03 | -.06 | .02 | -.05 | .04 | -.01 | -.14 | -.11 | -.15 | -.14 | -.17 | -.29 | - |  |  |  |  |  |  |  |  |
| Item 15 | .13 | -.05 | -.03 | .05 | -.07 | -.02 | .01 | .04 | -.09 | -.09 | -.18 | -.03 | -.28 | .48 | - |  |  |  |  |  |  |  |
| Item 16 | .20 | -.01 | .09 | .04 | -.10 | -.04 | -.03 | -.02 | -.14 | -.12 | -.22 | -.05 | -.24 | .39 | .75 | - |  |  |  |  |  |  |
| Item 17 | .03 | -.12 | -.12 | -.13 | -.22 | -.20 | -.11 | .02 | .01 | .03 | -.14 | .04 | -.14 | .31 | .41 | .42 | - |  |  |  |  |  |
| Item 18 | .04 | .00 | .06 | .09 | -.01 | -.06 | .00 | -.12 | -.18 | -.09 | -.13 | -.13 | -.23 | .23 | .36 | .49 | .23 | - |  |  |  |  |
| Item 19 | -.15 | -.14 | -.25 | -.20 | -.21 | -.22 | -.25 | -.22 | -.28 | -.21 | -.28 | -.28 | -.28 | .48 | .29 | .15 | .32 | .36 | - |  |  |  |
| *Factor 4* |  |  |  |  |  |  |  |  |  |  |  |  |  |  |  |  |  |  |  |  |  |  |
| Item 20 | .24 | .33 | .37 | .36 | .39 | .44 | .44 | .38 | .31 | .33 | .43 | .27 | .29 | -.10 | -.04 | .01 | -.23 | -.08 | -.28 | - |  |  |
| Item 21 | .32 | .44 | .34 | .36 | .38 | .46 | .41 | .30 | .32 | .32 | .45 | .33 | .29 | .01 | .04 | .08 | -.27 | -.11 | -.25 | .89 | - |  |
| Item 22 | .21 | .28 | .39 | .40 | .36 | .42 | .38 | .42 | .43 | .47 | .47 | .40 | .42 | -.06 | .01 | .02 | -.14 | -.11 | -.22 | .61 | .55 | - |
| Item 23 | .27 | .29 | .47 | .52 | .48 | .57 | .56 | .32 | .38 | .35 | .44 | .38 | .47 | .00 | .02 | -.02 | -.13 | -.10 | -.27 | .60 | .55 | .69 |

**Supplement D**

**Fragebogen zu Rollenkonflikten**

Nachfolgend finden Sie Einstellungen und Überzeugungen, die manche Dolmetschende im Rahmen der Tätigkeit haben. Bitte lesen Sie jede Aussage und schätzen Sie ein, wie stark dies auf Ihre Arbeit (z.B. im Bereich Psychotherapie) zutrifft. Hierbei gibt es keine richtigen oder falschen Antworten, allein Ihre Wahrnehmung ist wichtig.

*Das Wort „Auftraggebende“ kann durch einen Platzhalter ersetzt werden, wenn der Fragebogen für ein bestimmtes Arbeitssetting ausgefüllt wird (z.B. im Arbeitssetting Psychotherapie „Psychotherapeut*innen“ statt „Auftraggebende“).*

| 1  Trifft gar  nicht zu | 2 | 3 | 4  Trifft teilweise zu | 5 | 6 | 7  Trifft vollkommen zu |
| --- | --- | --- | --- | --- | --- | --- |

| **(Mangelnde) emotionale Abgrenzung** | | | | | | | |
| --- | --- | --- | --- | --- | --- | --- | --- |
| 1. Ich habe das Bedürfnis, Klient*innen während des Termins zu beruhigen. | 1 | 2 | 3 | 4 | 5 | 6 | 7 |
| 1. Ich muss während der Termine mit Klient*innen weinen. |  |  |  |  |  |  |  |
| 1. Ich muss nach den Terminen lange an die Klient*innen denken. |  |  |  |  |  |  |  |
| 1. Ich fühle mich nach den Terminen emotional belastet. |  |  |  |  |  |  |  |
| 1. Mir fällt es schwer, nach den Terminen von den Klient*innen gedanklich Abstand zu nehmen. |  |  |  |  |  |  |  |
| 1. Es fällt mir schwer, mich während des Termins emotional von Klient*innen abzugrenzen. |  |  |  |  |  |  |  |
| 1. Es fällt mir schwer, mich nach dem Termin emotional von Klient*innen abzugrenzen. |  |  |  |  |  |  |  |
| **Abwertung durch Auftraggebende** | | | | | | | |
| 1. Ich werde nicht als gleichwertige*r Kommunikationspartner*in von Auftraggebenden behandelt. |  |  |  |  |  |  |  |
| 1. Ich habe den Eindruck, dass Auftraggebende meine Arbeit unfair bewerten. |  |  |  |  |  |  |  |
| 1. Ich fühle mich von Auftraggebenden nicht wertgeschätzt. |  |  |  |  |  |  |  |
| 1. Ich habe den Eindruck, Auftraggebende sprechen in einem herabwürdigenden Ton mit mir. |  |  |  |  |  |  |  |
| 1. Ich habe den Eindruck, Auftraggebende sehen mich nur als technisches Werkzeug. |  |  |  |  |  |  |  |
| 1. Ich habe den Eindruck, Auftraggebende führen Missverständnisse auf schlechtes Dolmetschen zurück. |  |  |  |  |  |  |  |
| **Wahrgenommener formaler Rahmen der Dolmetschendenrolle** | | | | | | | |
| 1. Meine neutrale Rolle als Dolmetschende*r ist mit den kulturellen Werten der Klient*innen vereinbar. |  |  |  |  |  |  |  |
| 1. Meine Rolle als Dolmetschende*r ist klar durch Auftraggebende definiert. |  |  |  |  |  |  |  |
| 1. Meine Aufgaben als Dolmetschende*r sind klar von Auftraggebenden vorgegeben. |  |  |  |  |  |  |  |
| 1. Ich weiß, was Klient*innen von mir als Dolmetschende*r erwarten. |  |  |  |  |  |  |  |
| 1. Die Regeln zu meiner Dolmetschendentätigkeit sind mir von Auftraggebenden vermittelt worden. |  |  |  |  |  |  |  |
| 1. Klient*innen verstehen meine neutrale Rolle. |  |  |  |  |  |  |  |
| **Emotionale Belastung durch die Rolle innerhalb der Triade** | | | | | | | |
| 1. Ich habe Sorge, die Beziehung zwischen Klient*innen und Auftraggebenden zu stören. |  |  |  |  |  |  |  |
| 1. Ich habe Sorge, ein Hindernis für die Beziehung zwischen Klient*innen und Auftraggebenden zu sein. |  |  |  |  |  |  |  |
| 1. Ich habe Sorge, dass Klient*innen eine engere Beziehung zu mir haben als zu Auftraggebenden. |  |  |  |  |  |  |  |
| 1. Ich bin belastet, wenn es widersprüchliche Bedürfnisse von Klient*innen und Auftraggebenden während der Termine gibt. |  |  |  |  |  |  |  |

**Supplement E**

**CBI: subcale work-related exhaustion**

| **Eigen values** |
| --- |
| 3.96 |
| 0.85 |
| 0.64 |
| 0.52 |
| 0.39 |
| 0.36 |
| 0.28 |

Factor loadings:

|  | F1 |
| --- | --- |
| Item 1 | .81 |
| Item 2 | .86 |
| Item 3 | .84 |
| Item 4 | .46 |
| Item 5 | .78 |
| Item 6 | .77 |
| Item 7 | .87 |

Fit indices:

|  | 1-FaCtor |
| --- | --- |
| CFI | .951 |
| TLI | .926 |
| RMSEA | .103 |
| SRMR | .044 |

**ProQOL; subscale Secondary traumatic Stress**

| **Eigen values** |
| --- |
| 3.96 |
| 1.37 |
| 0.91 |
| 0.75 |
| 0.69 |
| 0.56 |
| 0.50 |
| 0.48 |
| 0.41 |
| 0.37 |

Factor loadings:

|  | 1-Factor | 2-Factor | |
| --- | --- | --- | --- |
|  | **F1** | **F1** | **F2** |
| Item 1 | .46 | .60 | -.23 |
| Item 2 | .47 | .49 | -.03 |
| Item 3 | .66 | .72 | -.07 |
| Item 4 | .77 | .74 | .07 |
| Item 5 | .76 | .65 | .21 |
| Item 6 | .66 | .73 | -.08 |
| Item 7 | .70 | .54 | .32 |
| Item 8 | .34 | -.03 | .71 |
| Item 9 | .55 | .30 | .49 |
| Item 10 | .10 | -.06 | .29 |

Fit indices:

|  | 1-FacTOR | 2-FacTOR |
| --- | --- | --- |
| CFI | .930 | 1.000 |
| TLI | .911 | 1.030 |
| RMSEA | .073 | 0.000 |
| SRMR | .067 | 0.030 |
